# BGP-15 Protects against Oxidative Stress- or Lipopolysaccharide-Induced Mitochondrial Destabilization and Reduces Mitochondrial Production of Reactive Oxygen Species

**DOI:** 10.1371/journal.pone.0169372

**Published:** 2017-01-03

**Authors:** Katalin Sumegi, Katalin Fekete, Csenge Antus, Balazs Debreceni, Eniko Hocsak, Ferenc Gallyas, Balazs Sumegi, Aliz Szabo

**Affiliations:** 1 Department of Biochemistry and Medical Chemistry, University of Pécs Medical School, Pécs, Hungary; 2 MTA-PTE Nuclear-Mitochondrial Interactions Research Group, University of Pécs Medical School, Pécs, Hungary; 3 Szentagothai Research Centre, Pécs, Hungary; University of South Alabama Mitchell Cancer Institute, UNITED STATES

## Abstract

Reactive oxygen species (ROS) play a critical role in the progression of mitochondria-related diseases. A novel insulin sensitizer drug candidate, BGP-15, has been shown to have protective effects in several oxidative stress-related diseases in animal and human studies. In this study, we investigated whether the protective effects of BGP-15 are predominantly via preserving mitochondrial integrity and reducing mitochondrial ROS production. BGP-15 was found to accumulate in the mitochondria, protect against ROS-induced mitochondrial depolarization and attenuate ROS-induced mitochondrial ROS production in a cell culture model, and also reduced ROS production predominantly at the complex I-III system in isolated mitochondria. At physiologically relevant concentrations, BGP-15 protected against hydrogen peroxide-induced cell death by reducing both apoptosis and necrosis. Additionally, it attenuated bacterial lipopolysaccharide (LPS)-induced collapse of mitochondrial membrane potential and ROS production in LPS-sensitive U-251 glioma cells, suggesting that BGP-15 may have a protective role in inflammatory diseases. However, BGP-15 did not have any antioxidant effects as shown by *in vitro* chemical and cell culture systems. These data suggest that BGP-15 could be a novel mitochondrial drug candidate for the prevention of ROS-related and inflammatory disease progression.

## Introduction

Mitochondria play a critical role in the progression of oxidative stress-related diseases, as excess mitochondrial metabolites cause mitochondrial hyperpolarization and production of reactive oxygen species (ROS) [1]. Extramitochondrial ROS can amplify mitochondrial ROS production leading to mitochondrial damage and cell death [2–4]. In addition, mutations in the mitochondrial genome can lead to excess ROS production, which promotes the development of several diseases, in addition to accelerated aging [5–8]. Mitochondrial membrane potential (ΔΨ) lowering agents such as uncouplers and ROS reducing agents (e.g. superoxide dismutase, N-acetylcysteine and lipoic acid) prevent the development of several diseases including hypertriglyceridemia, fatty liver disease, insulin resistance and sepsis [9–13]. However, antioxidant therapy has previously failed in human clinical studies despite the supporting cell culture and animal experiments [14,15]. This failure could be explained by the requirement for antioxidants to be present at millimolar concentrations in order to significantly decrease oxidative damage [16]. Furthermore, oxidative stress activates mitogen-activated protein kinase (MAPK) and tyrosine kinase signaling pathways [17,18], nuclear factor-κB (NF-κB), activator protein-1 (AP-1) and hypoxia-inducible factor-1 (HIF-1ɑ) transcription factors, cell cycle proteins [19] and ion channels [20,21]. Therefore, it may be more favorable to prevent those processes, which lead to disease-promoting alterations in the signaling pathways, rather than attempting to reduce ROS levels using antioxidants.

BGP-15 (O-[3-piperidino-2-hydroxy-1-propyl]-nicotinic amidoxime) has a wide range of cytoprotective effects [22–25], but up to now there is no information about its affecting a known receptor or specific binding protein. In disease model systems, it has been shown to protect cells against cell death [24,26] and increased heat shock protein (HSP) expression [22,27–29], in addition to inhibiting the nuclear translocation of apoptosis-inducing factor (AIF) from the mitochondria [24] and inhibits c-Jun N-terminal kinase (JNK) [23,29,30] and p38 MAPK [23] activation. It has also been shown that BGP-15 is an insulin sensitizer in an olanzapine-induced metabolic disorder in human phase II studies, and also in insulin-resistant patients [27,31,32]. This insulin sensitizing effect was suggested to be related to its HSP co-inducer effect [27,31,32], however, no molecular mechanism has been presented yet for the regulation of HSP expression by BGP-15.

Because of the importance of mitochondria in regulating cell death in ROS-related diseases [1,33], in this study, we investigated whether the protective effects of BGP-15 rely on the preservation of mitochondrial integrity and reduction of mitochondrial ROS production.

## Materials and Methods

### Materials

All chemicals for cell culture studies were purchased from PAA Laboratories (Cölbe, Germany) and Gibco/Invitrogen (Carlsbad, CA, USA). The fluorescent dyes JC-1, fluorescein-conjugated annexin V (annexin V-FITC), propidium iodide (PI), Hoechst 33342, rhodamine 123 (R123), dihydrorhodamine 123 (DHR123) and MitoSOX were obtained from Molecular Probes (Carlsbad, CA, USA). BGP-15 was a gift from N-Gene (New York, NY, USA). All remaining chemicals were purchased from Sigma Aldrich Co. (Budapest, Hungary). All reagents were of the highest purity commercially available.

### Cell cultures

WRL-68 (HeLa derivative), H9c2 (rat heart myoblast) and U-251 MG (human malignant glioblastoma; formerly known as U-373) cells were purchased from the European Collection of Cell Cultures. The cell lines were grown in a humidified 5% CO_2_ atmosphere at 37°C. WRL-68 cells were cultured in Eagle’s minimum essential medium, and H9c2 and U-251 MG cells in Dulbecco’s modified Eagle’s medium. All media contained an antibiotic solution (1% penicillin and streptomycin mixture) and 10% bovine serum. Cells were passaged every 3 days. Cells were seeded at a starting density of 2 × 10^4^ cells/well in a 96-well plate for the viability and ROS production assays, or at a density of 1 × 10^5^ cells/well in a 6-well plate for fluorescent microscopy.

### Animals

Wistar rats were purchased from Charles River Hungary Breeding Ltd. (Budapest, Hungary). The animals were kept under standard conditions and tap water and rat chow were provided *ad libitum*. The investigation conformed to the Guide for the Care and Use of Laboratory Animals, published by the US National Institutes of Health (NIH Publication no. 85–23, revised in 1996), and was approved by the Animal Research Review Committee of the University of Pécs, Hungary.

### Isolation of rat liver mitochondria

Rats were sacrificed by decapitation under isoflurane (AbbVie Ltd., Budapest, Hungary) anesthesia and mitochondria were isolated from the liver by differential centrifugation, as described in a standard protocol [34]. All isolated mitochondria were purified by Percoll density gradient centrifugation [35], and mitochondrial protein concentrations were determined using the biuret method with bovine serum albumin as a standard.

### Determination of membrane potential (ΔΨ) in isolated rat liver mitochondria

ΔΨ was monitored by measuring R123 fluorescence upon its release from the mitochondria. Fluorescence was measured using a fluorescence spectrometer (LS-50B; Perkin-Elmer; gift from Alexander von Humboldt Foundation, Bonn, Germany) at an excitation wavelength of 494 nm and an emission wavelength of 525 nm. Briefly, 1 mg protein/mL mitochondria were preincubated in assay buffer (70 mM sucrose, 214 mM mannitol, 20 mM N-2-hydroxyethyl piperazine-N′-2-ethanesulfonic acid, 5 mM glutamate, 0.5 mM malate and 0.5 mM phosphate) containing 1 μM R123 for 60 seconds. Alterations in ΔΨ were induced by the addition of BGP-15 at the indicated concentrations.

### Mitochondrial uptake of BGP-15

Mitochondrial uptake of BGP-15 (50 μM) was determined in 5 mM Tris buffer (pH 7.5) containing 150 mM KCl, 1 mM EDTA and 2.5 mg mitochondrial protein in 800 μl volume, in addition to 10 μM glucose-6-phosphate as a standard in order to determine the void volume. Uncoupling was induced with 50 μM 2,4-dinitrophenol. After an incubation of 10 min, the mitochondria was centrifuged at 20000 g, was washed then lysed in 800 μl of ethanol-water 1:1 solution, and centrifuged at 20000 g. Aliquots of the clear supernatant were freeze-dried, and taken up in aqueous formic acid solution (0.1%).

### HPLC-MS/MS analysis

Aliquots (5 μl) of the samples were injected into the HPLC-MS system (Dionex Ultimate 3000 UHPLC, Q-Exactive HRMS; Thermo Fisher Scientific, Bremen, Germany). Liquid chromatographic separation was carried out using a Kinetex (Gen-Lab Kft., Budapest, Hungary) 2.6 μm C18 100 Å HPLC column (100 × 2.1 mm), which was maintained at 30°C. The mobile phase used was (A) aqueous formic acid solution (0.1%) with (B) acetonitrilic formic acid solution (0.1%). The flow rate was set to 300 μL/minute. The initial gradient conditions were set to 5% B and held for 3 minutes, then B was increased linearly until it had reached 80% after 12 minutes. The initial conditions were reached after 2 minutes, then the column was equilibrated for 8 minutes. The mass spectrometer was equipped with a heated electrospray ion source which was operated in the negative ion mode. The spray voltage was set to 3.5 kV, the capillary temperature set to 300°C and the temperature of the probe heater was set to 50°C. The S-lens RF level was set to 70. Mass range was set to m/z 150–1500. Data analysis was performed using the Thermo Xcalibur (version 2.2 SP1.48) software. Ion intensities were determined by matching them to a BGP-15 calibration curve.

### Cell viability assay

The viability of WRL-68 cells after the different treatments were evaluated by sulforhodamine B (SRB) assay. The method is customarily used for cell density determination in cytotoxicity screening, based on the measurement of cellular protein content. The assay was performed according to the method described by Papazisis and colleagues [36], with some modifications. The culture medium was aspirated prior to fixation of the cells by the addition of 200 μL of cold 10% trichloroacetic acid. After a 20 minute incubation at 4°C, cells were washed twice with deionized water. Microplates were then left to dry at room temperature for at least 24 hours. When dried, the cells were stained with 200 μL of 0.1% SRB dissolved in 1% acetic acid for at least 20 minutes at room temperature, after which they were washed four times with 1% acetic acid to remove any unbound stain molecules. The plates were left to dry at room temperature. The bound SRB was solubilized with 200 μL of 10 mM unbuffered Tris-base solution and plates were left on a plate shaker for at least 10 minutes. Absorbance was measured using the GloMax Multi Detection System (Promega, USA) at 560 nm in addition to the background measurement at 620 nm. The optical density values were defined as the absorbance of each individual well minus the blank value (the mean optical density of the background control wells). All experiments were run in at least eight replicates and each measurement was repeated three times.

### Determination of reactive oxygen species in cell culture

Intracellular ROS (peroxinitrite, •OH and iron + hydrogen peroxide (H_2_O_2_)) were determined by using two separate approaches, fluorescence microscopy and quantitative determination of ROS-evoked fluorescence intensities by a plate reader. WRL-68 or U-251 MG cells were seeded to glass coverslips and cultured at least overnight before the experiment. WRL-68 cells were transiently transfected with mitochondria directed enhanced red fluorescent protein (mERFP). The next day, cells were washed twice with phosphate buffered saline (PBS), and were treated as it is described in the Results and the figure legends. Then, the medium was replaced to a fresh one containing 1 μM of the oxidation-sensitive DHR123 fluorescent dye, and the incubation continued for an additional 15 minutes to allow for oxidation of DHR123 by the endogenous ROS.

The fluorescence of mERFP and the oxidized DHR123 was excited at 615 and 485 nm and the evoked emission was measured at >650 and 525 nm, respectively using a Nikon Eclipse Ti-U fluorescent microscope (Auro-Science Consulting Ltd., Budapest, Hungary) equipped with a Spot RT3 camera using a 60x objective lens. The nuclei of U-251 MG cells were labelled using Hoechst 33342 (1 μg/mL) dye, which were excited at 350 nm and read at 460 nm emission wavelengths. All experiments were repeated three times.

Alternatively, WRL-68, H9c2 or U-251 MG cells were seeded in 96-wells plates and kept in Krebs-Henseleit buffer containing 10% fetal bovine serum (FBS).The WRL-68 and H9c2 cells were treated with either H_2_O_2_ for 30 minutes alone or with increasing concentrations of BGP-15 in the absence or presence of 20 μM MitoTEMPO (Sigma Aldrich Co.) After 30 minutes, DHR123 (1 μM) was added to the medium and R123 formation was detected after 15 minutes with the GloMax Multi Detection System (excitation wavelength was 490 nm and emission wavelength was between 510–570 nm). The U-251 MG cells were treated with either LPS for 30 minutes alone or with 50 μM BGP-15 in the absence or presence of 20 μM MitoTEMPO (Sigma Aldrich Co.). Superoxide anions were detected by the addition of MitoSOX (0.3 μM) fluorescent dye. Fluorescence of oxidized MitoSOX was excited at 365 nm and the evoked emission was measured at 410–460 nm by the GloMax Multi Detection System. All experiments were run in six replicates and each measurement was repeated three times.

### Determination of mitochondrial production of reactive oxygen species

Mitochondria (100 μg/mL) were dissolved in a buffer solution containing 20 mM 3-(N-morpholino)propanesulfonic acid (MOPS) and 314 mM sucrose (pH 7.4), which also contained either malate (5 mM) and glutamate (5 mM) or succinate (5 mM). ROS production was determined by the oxidation of DHR123 (1 μM) to R123, as measured by a fluorescence spectrometer at an excitation wavelength of 494 and an emission wavelength of 525 nm, under continuous mixing at 30°C. ROS production was localized to respiratory complexes by either blocking the electron flow with antimycin A (10 μM) where glutamate (5 mM) and malate (5 mM) were used as substrates in order to localize ROS production to complex I, or by blocking electron flow with potassium cyanide (1 mM) where succinate was used as the substrate to localize ROS production to complex III. The antioxidant capacities of BGP-15 were determined by the chemical oxidation of DHR123 (1 μM; excitation wavelength was 494 nm and emission wavelength was 525 nm) to R123. In these systems, we used either 500 μM H_2_O_2_ or 50 μM H_2_O_2_ plus 1 μM Fe^2+^-EDTA.

### Construction of mitochondria directed enhanced red fluorescent protein (mERFP; MitoRed)

The mERFP-expressing plasmid was constructed by firstly amplifying the mitochondrial targeting sequence by polymerase chain reaction from the cytochrome c oxidase subunit VIIIa (COX8A) gene (RZPD). The amplified sequence was then inserted into pDsRed-Monomer-N1 mammalian expression plasmid (Clontech-Takara Bio Europe; Saint-Germain-en-Laye, France) between the XhoI and HindIII restriction sites.

### JC-1 assay for fluorescent microscopy

ΔΨ was measured using the ΔΨ specific fluorescent probe, JC-1. WRL-68 or U-251 MG cells were seeded to glass coverslips and cultured at least overnight before the experiment. After the indicated treatment, cells were washed twice in ice-cold PBS, then incubated for 15 minutes at 37°C in modified Krebs-Henseleit solution containing 100 ng/mL of JC-1. When excited at 490 nm, the dye emits either green fluorescence at a low Δψ or red fluorescence at a high Δψ. Following incubation, the cells were washed once with modified Krebs-Henseleit solution, then visualized by a Nikon Eclipse Ti-U fluorescent microscope which was equipped with a Spot RT3 camera, using a 40x objective lens with epifluorescent illumination. All experiments were repeated three times.

### Tetramethylrhodamine methyl ester (TMRM) assay

WRL-68 cells were seeded in 96-wells plates, kept in Krebs-Henseleit buffer containing 10% fetal bovine serum (FBS), then treated with either H_2_O_2_ alone or with BGP-15 for 3 hours. In a separate experiment, U-251 MG cells were treated with either LPS for 60 minutes alone or with 50 μM BGP-15. Then, the medium was replaced with Krebs-Henseleit solution containing TMRM (50 nM), a cationic, cell-permeant, red fluorescent dye. After 15 minutes incubation, excess dye was washed off and the fluorescence was measured by the GloMax Multi Detection System (excitation wavelength was 525 nm and emission wavelength was between 580–640 nm). To assess aspecific adsorption of the dye, the fluorescence signal was remeasured after addition of 1 μM carbonyl cyanide 4-(trifluoromethoxy)phenylhydrazone (FCCP) a mitochondrial uncoupling agent. ΔΨ was calculated as the difference of fluoresescence signal before and after FCCP-treatment. All experiments were run in six replicates and the measurement was repeated three times.

### Identification of the type of cell death by annexin V/PI staining

Cell death was detected by the GloMax Multi Detection System after annexin V-FITC/PI double staining. WRL-68 cells were seeded at 2 × 10^4^cells/well in a 96-well, then cultured at least overnight before the experiment. After subjecting the cells to the indicated treatment, cells were washed once in PBS and then incubated in modified Krebs-Henseleit solution containing FITC-conjugated annexin V and PI, according to the manufacturer’s protocol. Following incubation, the cells were washed once with modified Krebs-Henseleit solution. Then green fluorescence signal (annexin V-FITC) was detected with the GloMax Multi Detection System (excitation wavelength was 490 nm and emission wavelength was 518 nm). The red fluorescence signal (PI) was excited at 525 nm and the evoked emission was measured at 617 nm. All experiments were run in six replicates and each measurement was repeated three times.

### Statistical analyses

All data were expressed as the mean ± standard error of mean (SEM) of the replicate measurements. Differences between treatment groups were determined by ANOVA with a post-hoc test. A Student’s t-test was used to compare the mean values from the two groups. Differences were regarded as significant when the P-value was < 0.05.

## Results

### Mitochondrial uptake of BGP-15

**Fig 1:**
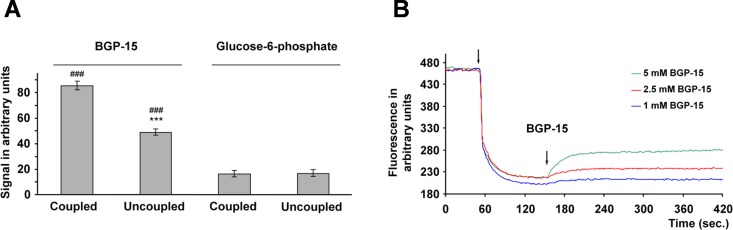
BGP-15 is enriched in mitochondria and reduces membrane potential (ΔΨ) in isolated mitochondria. (A) Membrane potential enhanced the mitochondrial uptake of BGP-15 (50 μM) in isolated rat liver mitochondria. Uncoupling was found to occur with 50 μM 2,4-dinitrophenol. Data are presented as the mean ± SEM of three independent experiments. ***P < 0.001 compared to coupled mitochondria, ^###^P < 0.001 compared to the glucose-6-phosphate signal. (B) Mitochondrial membrane potential was monitored by measuring the fluorescence intensity of R123, a cationic fluorescent dye. Isolated rat liver mitochondria, represented by the first arrow, took up the dye in a voltage-dependent manner, resulting in fluorescent quenching. At the second arrow either 1 mM, 2.5 mM or 5 mM BGP-15 was added. A representative plot of three independent concurrent experiments is presented.

BGP-15 possesses a delocalized positive charge, therefore, it is suitable for determining membrane potential-dependent uptake. Accordingly, we measured BGP-15 uptake in both energized and uncoupled mitochondria. The void volume was determined using glucose-6-phosphate, a substance that cannot permeate the mitochondrial inner membrane. When incubated in the presence of 50 μM BGP-15 for 10 minutes, the energized mitochondria took up more than 85% of the drug (Fig 1A), suggesting that the majority of BGP-15 was taken up in a membrane potential-dependent manner. Complete uncoupling by dinitrophenol significantly decreased BGP-15 uptake (Fig 1A). However, even the uncoupled mitochondria were found to bind more BGP-15 than the amount corresponding to the void volume, indicating that BGP-15 interacted with the mitochondrial proteins and/or lipids. Extrapolating this finding to physiological conditions, it is likely that more than 90% of BGP-15 had accumulated in the mitochondria, which raises the possibility that BGP-15 may protect cells via mitochondrial mechanisms.

### Effect of BGP-15 on mitochondrial membrane potential (ΔΨ)

Since mild uncoupling of the mitochondria could be beneficial in insulin resistance [37], we tested the effect of BGP-15, an insulin sensitizer [27,31,32] on Δψ by using a Δψ-sensitive dye (R123) in isolated rat liver mitochondria. Treatment with BGP-15 alone resulted in a concentration-dependent decrease in Δψ at millimolar concentrations (Fig 1B). The effect on Δψ of submillimolar concentrations of BGP-15 was below the detection limit (Fig 1B) suggesting that at the 50 μM concentration we have used throughout the study the drug could hardly cause any mitochondrial depolarization.

**Fig 2:**
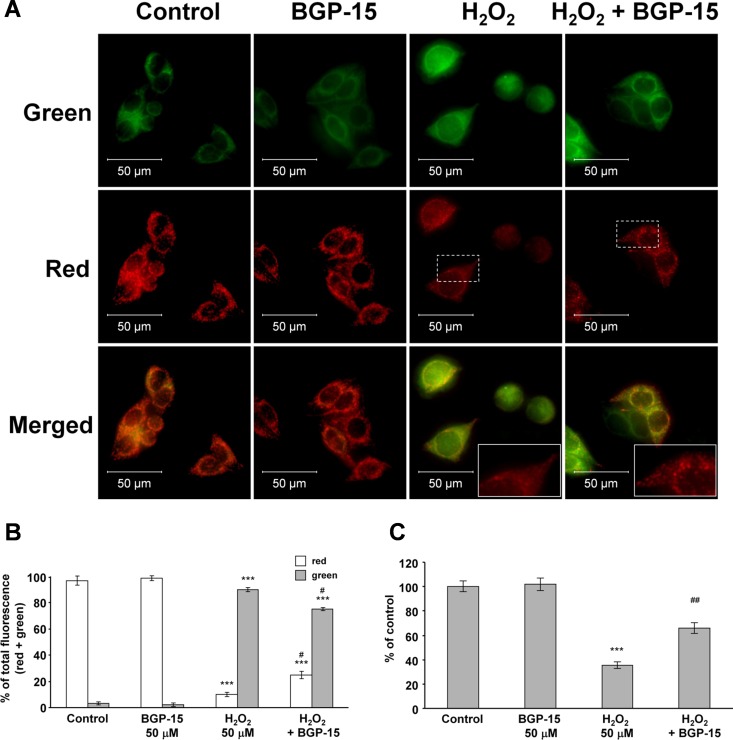
BGP-15 protects against reactive oxygen species-induced depolarization of mitochondria in WRL-68 cells, as determined by JC-1 and TMRM. (A) Effect of BGP-15 on H_2_O_2_-induced mitochondrial membrane depolarization in WRL-68 cells. Cells were exposed to 50 μM H_2_O_2_ in the absence or presence of 50 μM BGP-15 for 3 hours, then stained with 100 ng/mL of JC-1, a membrane potential-sensitive fluorescent dye. The dye was loaded, and after a 15 minute incubation fluorescent microscopic images were taken using both the red and green channels. The inserts show the homogenous red fluorescence in H_2_O_2_-treated cells, and the dotted labelling represents the H_2_O_2_ + BGP-15 treated cells, showing that BGP-15 protected the mitochondrial integrity in the presence of H_2_O_2_. Inserts are expanded from the area indicated by dashed rectangles. Representative merged images of three independent experiments are presented. (B) Quantitative analysis of mitochondrial depolarization induced by H_2_O_2_ (50 μM) and its reduction by BGP-15 (50 μM) in WRL-68 cells. Results are presented as the mean ± SEM. ***P < 0.001 compared to control cells, ^#^P < 0.05 compared to H_2_O_2_-treated cells. (C) Effect of BGP-15 on H_2_O_2_-induced mitochondrial membrane depolarization in WRL-68 cells. Cells were treated with 50 μM H_2_O_2_ in the absence or presence of 50 μM BGP-15 for 3 hours, then stained with 50 ng/mL of TMRM, a cationic, cell-permeant, red fluorescent dye. After a 15 minutes incubation fluorescent signal was measured by the GloMax Multi Detection System, then remeasured after the application of 1 μM FCCP ΔΨ was calculated as the difference of fluoresescence signal before and after FCCP-treatment. Data are presented as the mean ± SEM of three independent experiments. **P < 0.01, ***P < 0.001 compared to control cells; ^##^P < 0.01 compared to H_2_O_2_-treated cells.

Under cell culture conditions, we analyzed the effect of BGP-15 on Δψ using JC-1, a cell-permeable voltage-sensitive fluorescent mitochondrial dye. JC-1 emits red fluorescence in highly energized mitochondria (aggregated dye), while depolarized mitochondria emit green fluorescence (monomer dye). WRL-68 cells were incubated in the presence of 50 μM H_2_O_2_, either alone or together with 50 μM BGP-15, for 3 hours before loading with 100 ng/mL JC-1 dye for 15 minutes, after which fluorescent microscopy was performed. In the control and BGP-15-treated cells, fluorescence microscopy showed strong red fluorescence and weak green fluorescence, which indicates a high ΔΨ in mitochondria (Fig 2A and 2B). The addition of H_2_O_2_ to cells facilitates the depolarization of mitochondria, resulting in weaker red fluorescence and stronger green fluorescence (Fig 2A and 2B). When H_2_O_2_ was added to cells in addition to BGP-15, the depolarization of mitochondria was found to be weaker, as shown by a smaller decrease in red fluorescence and weaker increase in green fluorescence (Fig 2A and 2B). This was also demonstrated by the accurate (mitochondrial) labelling of red fluorescence in the cells treated with both H_2_O_2_ and BGP-15, and in cells treated with H_2_O_2_ only (Fig 2A). The quantitative assessment revealed that BGP-15 did not affect the ΔΨ at a concentration of 50 μM (Fig 2B); however, it was found to reduce the H_2_O_2_-induced depolarization of the mitochondrial membrane (Fig 2B), suggesting that even at this concentration it protected the ΔΨ against oxidative stress. We have obtained identical results when we assessed ΔΨ by using TMRM, another membrane potential sensitive fluorescent dye and a quantitative, plate reader-based method (Fig 2C).

### BGP-15 attenuates mitochondrial production of reactive oxygen species

**Fig 3:**
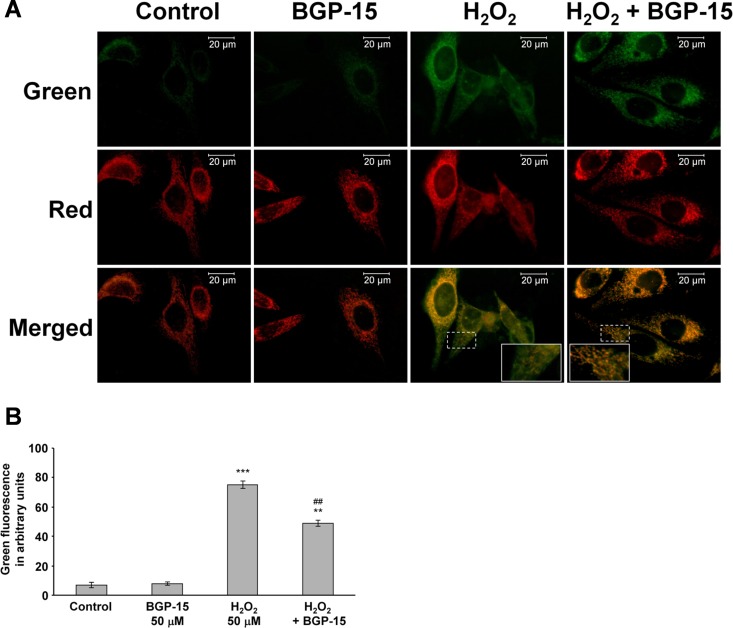
BGP-15 attenuates hydrogen peroxide-induced mitochondrial reactive oxygen species production in WRL-68 cells. (A) Effect of hydrogen peroxide and BGP-15 pretreatment (for 30 minutes) on mitochondrial ROS production, as determined by the oxidation of the mitochondrial enriched dye from DHR123 to R123 in WRL-68 cells that had been labelled with mitochondrial directed red fluorescent protein. High magnification fluorescent microscopic images show the different localization of the produced R123. Inserts are expanded from the area indicated by dashed rectangles. (B) Quantification of R123 production. Data are presented as the mean ± SEM of three independent experiments. **P < 0.01 and ***P < 0.001 compared to control cells; ^##^P < 0.01 compared to H_2_O_2_-treated cells.

Destabilization of the mitochondrial membrane systems has previously been reported to contribute to mitochondrial ROS production [38]. As BGP-15 was shown to protect the mitochondrial membrane system under oxidative stress (Fig 2A, 2B and 2C), we assumed that it could also affect mitochondrial ROS production. To investigate this possibility, WRL-68 cells were transfected with mERFP to label the mitochondria, and then incubated in the presence or absence of 50 μM H_2_O_2_ for 30 minutes. Fresh medium containing DHR123 was added, and after 15 minutes the amount of green R123 fluorescence, as a result of oxidation from non-fluorescent DHR123, was measured by fluorescence microscopy. BGP-15 at a concentration of 50 μM did not affect the weak green fluorescence of mitochondrial localization (Fig 3A). However, its addition did reduce the substantial increase in green fluorescence induced by the H_2_O_2_ treatment (Fig 3A and 3B). Furthermore, oxidative stress-induced green fluorescence was diffuse in the absence of BGP-15, whereas it was predominantly localized to the mitochondria in the presence of BGP-15 (Fig 3A inserts). Although the R123 fluorescence could result from cytosolicly produced R123 taken up to the mitochondria and DHR123 oxidized by mitochondrial ROS, we think that R123 fluorescence reflected mitochondrial ROS production, as it was predominantly localized to the mitochondria when Δψ was not compromised.

**Fig 4:**
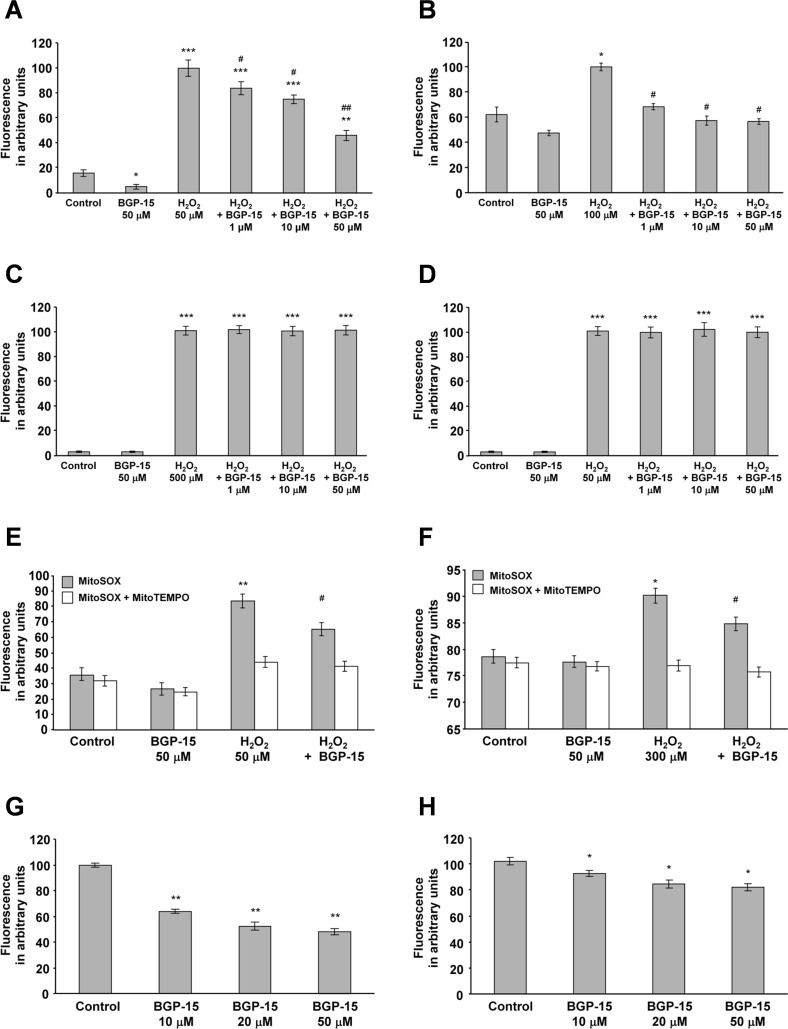
BGP-15 attenuates oxidative stress-induced DHR123 oxidation and superoxide formation in complexes I-III in WRL-68 and H9c2 cells. (A) Effect of BGP-15 on oxidative stress-induced DHR123 oxidation in WRL-68 cells. Data are presented as the mean ± SEM of three independent experiments. *P < 0.05, **P < 0.01 and ***P < 0.001 compared to control cells; ^#^P < 0.05 and ^##^P < 0.01 compared to H_2_O_2_-treated cells. (B) Effect of BGP-15 on oxidative stress-induced DHR123 oxidation in H9c2 cardiomyocytes. Results are presented as the mean ± SEM of three independent experiments. *P < 0.05 compared to control cells, ^#^P < 0.05 compared to H_2_O_2_-treated cells. (C) BGP-15 in chemical reactions does not inhibit DHR123 oxidation induced by H_2_O_2_ (500 μM). Data are presented as the mean ± SEM of three independent experiments. ***P < 0.001 compared to control group. (D) BGP-15 in chemical reactions does not inhibit DHR123 oxidation induced by H_2_O_2_ (50 μM) and Fe(II)-EDTA (66 μM) (Fenton reaction system). Results are presented as the mean ± SEM of three independent experiments. ***P < 0.001 compared to the control group. (E) Effect of BGP-15 on oxidative stress-induced superoxide production in WRL-68 cells in the absence or presence of 20 μM MitoTEMPO as determined by MitoSOX (0.3 μM). Data are presented as the mean ± SEM of three independent experiments. *P < 0.05, **P < 0.01 compared to control cells, ^#^P < 0.05 compared to H_2_O_2_-treated cells. (F) Effect of BGP-15 on the oxidative stress-induced superoxide production in H9c2 cardiomyocytes in the absence or presence of 20 μM MitoTEMPO as determined by MitoSOX (0.3 μM). Results are presented as the mean ± SEM of three independent experiments. *P < 0.05 compared to control cells, ^#^P < 0.05 compared to H_2_O_2_-treated cells. (G) Effect of BGP-15 on mitochondrial DHR123 oxidation using glutamate-malate as substrate and with complex III inhibited by antimycin A. Data are presented as the mean ± SEM of three independent experiments. **P < 0.01 compared to the control group. (H) Effect of BGP-15 on mitochondrial DHR123 oxidation using succinate as substrate and with complex IV inhibited by CN^-^. Results are presented as the mean ± SEM of three independent experiments. *P < 0.05 compared to the control group.

As found previously (N-Gene Inc., personal communication), BGP-15 serum concentration does not exceed 25 nmoles/mL. Therefore, we analyzed the effect of BGP-15 on ROS-induced ROS production at concentrations ranging between 1–50 μM under cell culture conditions using a quantitative, plate-reader based method instead of microscopy. BGP-15 had a concentration-dependent inhibitory effect on the ROS-induced ROS production in WRL-68 cells, which was significant even at the 1 μM concentration (Fig 4A). In order to show that these observations apply to other cell lines too, we analyzed the effect of BGP-15 on H_2_O_2_-induced ROS production in H9c2 cardiomyocytes using the same system. Fig 4B shows that BGP-15 decreased the ROS-induced ROS production in H9c2 cardiomyocytes in a concentration-dependent manner (1–50 μM range).

In order to study whether the observed antioxidant effect of BGP-15 have resulted from scavenging property of the drug, we determined its effect on 500 μM H_2_O_2_- (Fig 4C) and H_2_O_2_ and Fe^2+^-EDTA (Fenton reaction, Fig 4D)-induced DHR123 oxidation in cell-free systems. Since BGP-15 did not affect DHR123 oxidation in any of these systems (Fig 4C and 4D) its antioxidant effect could not resulted from scavenging property of the drug. DHR123 can be oxidized predominantly by peroxynitrite and hydroxyl radicals that are formed in the Fenton reaction; however, it cannot detect superoxide. Therefore, we wanted to investigate whether BGP-15 could reduce the production of mitochondrial superoxide. To this end, we determined H_2_O_2_-induced ROS production in WRL-68 (Fig 4E) and H9c2 (Fig 4F) cells using the mitochondria-targeted redox fluorescent dye MitoSOX [39] instead of DHR123. Essentially, the results were comparable to those we obtained with DHR123 (Fig 4A and 4B vs. 4E and 4F). Furthermore, we repeated these experiments in the presence of the mitochondria-targeted antioxidant MitoTEMPO [40]. MitoTEMPO abolished H_2_O_2_-induced ROS production in all groups (Fig 4E and 4F) indicating mitochondrial localization of the BGP-sensitive ROS production. These data provide evidence that BGP-15 reduced mitochondrial superoxide production in both WRL-68 cells (Fig 4E) and H9c2 cardiomyocytes (Fig 4F).

### Effect of BGP-15 on mitochondrial reactive oxygen species production in isolated mitochondria

In order to provide unequivocal evidence for the mitochondrial mechanism underlying the inhibitory effect of BGP-15 on ROS-induced ROS production, we used Percoll gradient-purified mitochondria. We determined the effect of BGP-15 on the oxidation of DHR123 in the presence of glutamate and malate as substrates, with antimycin A for complex III inhibition, and showed the production of ROS in complex I and the complex III cytochrome b region of the respiratory chain. We found that the addition of BGP-15 at concentrations of 10 to 50 μM had a ~50% inhibitory effect on mitochondrial ROS production (Fig 4G). These results suggest that the target of BGP-15 was between complex I and the complex III cytochrome b region of the respiratory chain [41].

BGP-15 was also shown to reduce mitochondrial ROS production in the presence of succinate as a substrate and CN^-^ as the cytochrome oxidase inhibitor (Fig 4H), however, to a much smaller extent. This suggests that BGP-15 affected ROS production mainly via complex I and the cytochrome b part of complex III. These data show that BGP-15 has a specific inhibitory effect on mitochondrial ROS production at the complex I and complex III cytochrome b region of the respiratory chain, which is not an antioxidant effect.

### Effect of BGP-15 on reactive oxygen species-induced cell death

**Fig 5:**
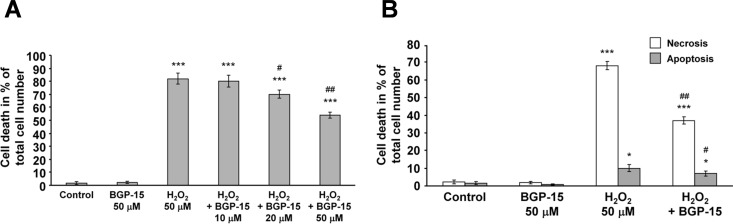
BGP-15 attenuates reactive oxygen species-induced apoptotic and necrotic cell death in WRL-68 cells. (A) BGP-15 protects against H_2_O_2_-induced cell death (sulforhodamine B assay). Data are presented as the mean ± SEM of eight independent experiments. ***P < 0.001 compared to control cells; ^#^P < 0.05 and ^##^P < 0.01 compared to H_2_O_2_-treated cells. (B) Determination of the effect of BGP-15 on H_2_O_2_-induced apoptosis (fluorescein-labelled annexin V) and necrosis (propidium iodide) pathways. Data are presented as the mean ± SEM of three independent experiments. *P < 0.05 and ***P < 0.001 compared to control cells; ^#^P < 0.05 and ^##^P < 0.01 compared to H_2_O_2_-treated cells.

As BGP-15 was shown to protect against H_2_O_2_-induced mitochondrial damage in addition to reducing mitochondrial ROS production, we analyzed its effects on H_2_O_2_-induced cell death. We treated WRL-68 cells with 50 μM H_2_O_2_ in the presence of 0–50 μM BGP-15 for 24 hours, then determined cell survival using the SRB method. We found that, consistent with its aforementioned protective effects (Figs 2–4), BGP-15 increased cell survival in a concentration-dependent manner (Fig 5A). To further investigate the underlying mechanism behind the cytoprotective effect of BGP-15, we determined the proportion of apoptosis and necrosis using annexin V-conjugated fluorescein-isothiocyanate and PI staining, which was performed after exposing the cells to 50 μM H_2_O_2_ in the presence or absence of 50 μM BGP-15 for 24 hours, which was then measured by the GloMax Multi Detection System. We found that under these conditions, H_2_O_2_-induced cell death was predominantly necrotic and only approximately 10% of the cells died by apoptosis (Fig 5B). BGP-15 significantly reduced both apoptotic and necrotic cell death, which was likely to be a result of mitochondrial protection.

### BGP-15 protects against LPS-induced mitochondrial depolarization

**Fig 6:**
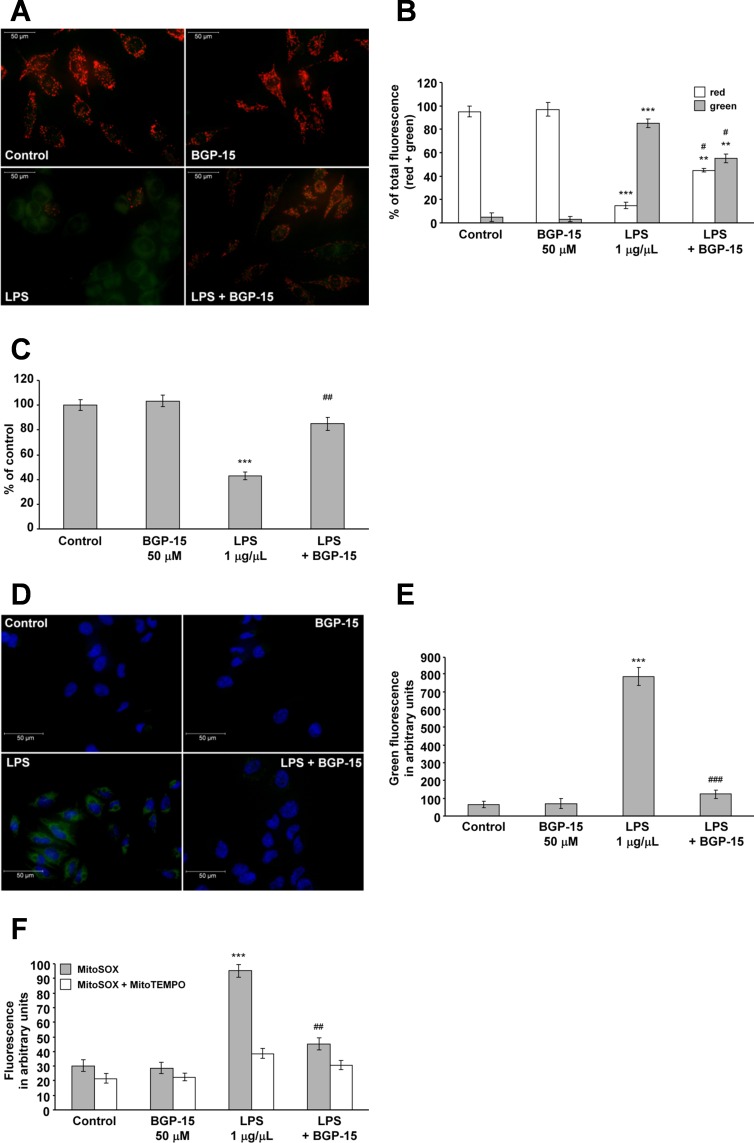
BGP-15 attenuates lipopolysaccharide-induced mitochondrial depolarization and production of reactive oxygen species. (A) Effect of BGP-15 on LPS-induced mitochondrial membrane depolarization in U-251 MG cells. Cells were exposed to 1 μg/mL LPS in the absence or presence of 50 μM BGP-15 for 1 hour, then stained with 100 ng/mL of JC-1. Fluorescent microscopic images were taken using both the red and green channels. Representative merged images of three independent experiments are presented. (B) Quantitative analysis of LPS-induced (1 μg/mL) mitochondrial depolarization and its reduction by BGP-15 (50 μM) in U-251 MG cells. Results are presented as the mean ± SEM. **P < 0.01 and ***P < 0.001 compared to control cells; ^#^P < 0.05 compared to LPS-treated cells. (C) Effect of BGP-15 on LPS-induced mitochondrial membrane depolarization in U-251 MG cells. Cells were treated with 1 μg/mL LPS in the absence or presence of 50 μM BGP-15 for 1 hour, then stained with 50 nM of TMRM. After a 15 minutes incubation fluorescent signal was measured by the GloMax Multi Detection System, then remeasured after the application of 1 μM FCCP. ΔΨ was calculated as the difference of fluoresescence signal before and after FCCP-treatment. Data are presented as the mean ± SEM of three independent experiments. *P < 0.05, ***P < 0.001 compared to control cells; ^##^P < 0.01 compared to LPS-treated cells. (D) Effect of BGP-15 on the LPS-induced ROS production in U-251 MG cells (containing the TLR4 receptor). Cells were treated with 1 mg/mL LPS in the presence or absence of 50 μM BGP-15 for 30 minutes. LPS-induced ROS production was determined by the oxidation of DHR123 (1 μM) to R123, measured with fluorescent microscopy. Cell nuclei were labelled using Hoechst 33342. Representative merged images of three independent experiments are presented. (E) Quantitative analysis of LPS-induced (1 μg/mL) ROS production and the protective effect of BGP-15 (50 μM). Data are presented as the mean ± SEM of three independent experiments. ***P < 0.001 compared to control cells; ^###^P < 0.001 compared to LPS-treated cells. (F) Effect of BGP-15 on oxidative stress-induced superoxide production in U-251 MG cells in the absence or presence of 20 μM MitoTEMPO as determined by MitoSOX (0.3 μM). Data are presented as the mean ± SEM of three independent experiments. *P < 0.05, ***P < 0.001 compared to control cells, ^##^P < 0.01 compared to LPS-treated cells.

It has been shown that the U-251 MG human malignant glioblastoma cell line contains components of the LPS signaling pathway [42], and LPS-induced signaling has been shown to be important in neurodegenerative, liver and several other diseases [43,44]. Additionally, mitochondria have been reported to play an important role in LPS signaling [43–47]. Therefore, we analyzed the effect of BGP-15 on LPS-induced mitochondrial depolarization in the U-251 MG cell line. This system is much more complex than the ROS-induced depolarization because LPS induces toll-like receptor 4 (TLR4)-dependent signaling and NADPH oxidase (NOX)-dependent ROS production, thereby increasing intracellular Ca^2+^ levels [48,49]. Mitochondrial depolarization was found to be induced by the addition of 1 μg/mL LPS for 1 hour, as determined by JC-1 staining and fluorescent microscopy (Fig 6A and 6B). We found that the addition of BGP-15 alone did not affect Δψ, but significantly attenuated LPS-induced mitochondrial depolarization in the U-251 MG human malignant glioblastoma cells (Fig 6A and 6B). We have obtained identical results when we assessed ΔΨ by using TMRM, another membrane potential sensitive fluorescent dye and a quantitative, plate reader-based method (Fig 6C). These data suggest that BGP-15 may play a role in inflammatory processes by a novel mitochondrial mechanism.

### BGP-15 protects against LPS-induced production of reactive oxygen species

Due to the association between mitochondrial depolarization and ROS production [38], we measured LPS-induced ROS production in the U-251 MG cells. We exposed the cells to 1 μg/mL LPS in the presence or absence of 50 μM BGP-15 for 1 hour, and then measured the fluorescence of R123 which had been oxidized from non-fluorescent DHR123 by the ROS (Fig 6D and 6E). Very low fluorescence intensities were detected in the untreated and BGP-15-treated cells, however, the addition of LPS was found to greatly induce ROS production (Fig 6D and 6E). BGP-15 reduced the LPS-induced ROS production almost to control levels (Fig 6D and 6E). Similarly to the H_2_O_2_-induced ROS production, we wanted to determine the intracellular localization of the LPS-induced ROS. To this end, we repeated the previous experiment using MitoSOX instead of DHR123 and a plate-reader instead of microscopy. Essentially, the results were comparable to those we obtained with DHR123 (Fig 6D and 6E vs. 6F). Furthermore, we repeated the experiment in the presence of MitoTEMPO. MitoTEMPO abolished LPS-induced ROS production (Fig 6F) indicating mitochondrial localization of the BGP-sensitive ROS production. These data provide evidence that BGP-15 reduced LPS-induced mitochondrial superoxide production in.

## Discussion

BGP-15 has been shown to have a protective effect in several disease models, including ischemic heart disease [30], Duchenne muscular dystrophy [22], neuropathy [25], cisplatin-induced kidney disease [26], glivec-induced cardiac disease [23] and paracetamol-induced liver disease [24], in addition to insulin resistance, as investigated in both animal and human studies [27,31,32]. For all these diseases, oxidative stress and inflammatory processes play an important role in disease progression, and in several cases, mitochondrial damage is pivotal. Therefore, we studied the effect of BGP-15 on ROS- or inflammatory response-induced mitochondrial damage in cell culture models. We focused on the effects of BGP-15 on mitochondrial membrane stability and ROS production, which are critical for mitochondrial-induced signaling, energy metabolism and mitochondrial cell death pathways. Recently, it has been shown that mild mitochondrial uncoupling can be protective in several disease models, including insulin resistance [37], hypertriglyceridemia and fatty liver disease [9], and can have a regulatory role in endocrine cross-talk via the induction of fibroblast growth factor 21 and the growth hormone/insulin-like growth factor I axis [10]. However, as we found, BGP-15 exerted a mild uncoupling effect only in millimolar concentrations. Since we have used BGP-15 at a 50 μM concentration, it is unlikely that its uncoupling effect played a significant role in its mitochondria- and cytoprotecting effects. On the other hand, BGP-15 reduced the oxidative stress-induced mitochondrial depolarization as we revealed by using two membrane potential sensitive dyes. ROS-induced mitochondrial depolarization could result in decreased ATP synthesis, increased superoxide production by the electron-transport chain, release of proapoptotic proteins from the intermembrane space, decreased mitochondrial fusion and increased fission [33,50–52]. All of these processes cause disturbance of cellular energy metabolism and a shift toward proapoptotic signaling eventually resulting in death of the cell [33,50–52]. Therefore, the membrane potential stabilizing effect of BGP-15 likely contributed to its protective effect in the aforementioned diseases.

BGP-15 was also found to be critical in the reduction of ROS- and LPS-induced ROS production (Figs 3A and 6D), which can play a significant role in the progression of several diseases. However, it is difficult to localize the site of ROS production by using conventional mitochondria-targeted redox dyes since oxidation of the dye by extramitochondrial or mitochondrial ROS results in identical fluorescence localized to the mitochondria. Therefore, we used MitoSOX [39] at a concentration of 0.3 μM excited at 365 nm. Under these conditions, the resulting 440 nm fluorescence is believed to result from oxidation of the dye by mitochondrially produced superoxide (Fig 4E and 4F). Furthermore, we have quenched mitochondrial ROS by the mitochondria-targeted antioxidant MitoTEMPO [40]. All the results supported that BGP-15 reduced mitochondrial ROS production. This effect could not result from antioxidant property of the molecule as it was revealed by our experiments on various cell-free ROS-generating systems (Fig 4C and 4D). Even, we believe that we localized the most important target of BGP-15, complex I-III, which is critical for ROS production by the respiratory chain. It can generate significant amounts of ROS under conditions of hypoxia, mitochondrial hyperpolarization, inhibition of respiratory complexes and in several other conditions [53]. The significant ROS-reducing effect of BGP-15 may be important in regulating ROS-dependent processes including cell death, MAPK and poly(ADP-ribose) polymerase (PARP) pathways, in addition to transcription factor activation (NF-κB, AP-1, NRF2, etc.) [54]. The high enrichment of BGP-15 in the mitochondria, combined with the significant reduction in mitochondrial ROS production at complex I by BGP-15, suggests that the protective effect of BGP-15 observed in different disease models is likely to be mediated by the aforementioned mitochondrial mechanisms. Our results also suggest that the effect of BGP-15 on signaling pathways, such as BGP-15-induced reduction in JNK and p38 MAPK activation [55] or BGP-15-induced Akt activation [56], could also potentially be related to reduced ROS production. LPS induces a complex stress pattern in sensitive cells, including ROS production by NADPH oxidases, an increase in cytoplasmic free calcium level and activation of mitochondria damaging signaling pathways [57,58]. In a separate study on cyclophilin D (CypD) knockout mice, we reported that LPS-induced mitochondrial ROS production was substantially reduced in CypD deficient cells and tissues, accompanied by reduced MAPK activation [45,46]. This stabilized the mitochondrial membrane systems by preventing high calcium-induced mitochondrial permeability transition, resulting in attenuated ROS production and benign intracellular signaling. Therefore, we assumed that, similar to the oxidative stress situation, BGP-15 would protect against LPS-induced mitochondrial damage. Indeed, we found that BGP-15 prevented LPS-induced mitochondrial depolarization and ROS production (Fig 6), demonstrating that BGP-15 can protect mitochondria against complex inflammatory damage as well as against ROS-induced damage. These results suggest the potential of BGP-15 as an experimental drug, not only in ROS-related diseases, but also in inflammatory diseases.

**Fig 7:**
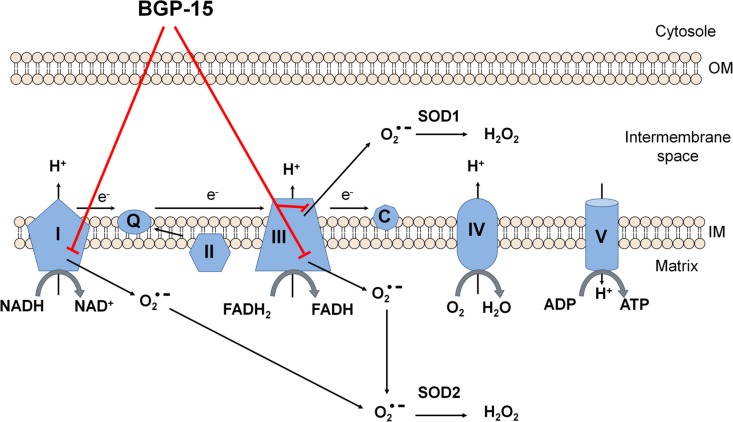
Possible mitochondrial molecular mechanism of BGP-15 cytoprotective action. BGP-15 reduces mitochondrial ROS production at complex I and at complex III, and so reduces ROS induced mitochondrial damage, as well as cell death.

In conclusion, the critical mechanism underlying the protective effect of BGP-15 on the mitochondria appear to be due to reduced ROS production, predominantly at the first and third respiratory complexes (Fig 7). By this mechanism, it may regulate several pathways that play critical roles in the progression of ROS-related and inflammatory diseases.

## References

[1] Wang W, Gong G, Wang X, Wei-LaPierre L, Cheng H, Dirksen R, et al (2016) Mitochondrial Flash: Integrative Reactive Oxygen Species and pH Signals in Cell and Organelle Biology. Antioxid Redox Signal 25: 534–549. 10.1089/ars.2016.6739 27245241 PMC5035371

[2] Daiber A, Di Lisa F, Oelze M, Kroller-Schon S, Steven S, Schulz E, et al (2015) Crosstalk of mitochondria with NADPH oxidase via reactive oxygen and nitrogen species signalling and its role for vascular function. Br J Pharmacol. 10.1111/bph.13403 PMC5446573 26660451

[3] Dan Dunn J, Alvarez LA, Zhang X, Soldati T (2015) Reactive oxygen species and mitochondria: A nexus of cellular homeostasis. Redox Biol 6: 472–485. 10.1016/j.redox.2015.09.005 26432659 PMC4596921

[4] Tajeddine N (2016) How do reactive oxygen species and calcium trigger mitochondrial membrane permeabilisation? Biochim Biophys Acta 1860: 1079–1088. 10.1016/j.bbagen.2016.02.013 26922832

[5] Baines HL, Turnbull DM, Greaves LC (2014) Human stem cell aging: do mitochondrial DNA mutations have a causal role? Aging Cell 13: 201–205. 10.1111/acel.12199 24382254 PMC4331785

[6] Szczepanowska K, Trifunovic A (2015) Different faces of mitochondrial DNA mutators. Biochim Biophys Acta 1847: 1362–1372. 10.1016/j.bbabio.2015.05.016 26014346

[7] Mazzaccara C, Iafusco D, Liguori R, Ferrigno M, Galderisi A, Vitale D, et al (2012) Mitochondrial diabetes in children: seek and you will find it. PLoS One 7: e34956 10.1371/journal.pone.0034956 22536343 PMC3334935

[8] Schapira AH (2012) Mitochondrial diseases. Lancet 379: 1825–1834. 10.1016/S0140-6736(11)61305-6 22482939

[9] Perry RJ, Kim T, Zhang XM, Lee HY, Pesta D, Popov VB, et al (2013) Reversal of hypertriglyceridemia, fatty liver disease, and insulin resistance by a liver-targeted mitochondrial uncoupler. Cell Metab 18: 740–748. 10.1016/j.cmet.2013.10.004 24206666 PMC4104686

[10] Keipert S, Ost M, Johann K, Imber F, Jastroch M, van Schothorst EM, et al (2014) Skeletal muscle mitochondrial uncoupling drives endocrine cross-talk through the induction of FGF21 as a myokine. Am J Physiol Endocrinol Metab 306: E469–482. 10.1152/ajpendo.00330.2013 24347058

[11] Bavarsad Shahripour R, Harrigan MR, Alexandrov AV (2014) N-acetylcysteine (NAC) in neurological disorders: mechanisms of action and therapeutic opportunities. Brain Behav 4: 108–122. 10.1002/brb3.208 24683506 PMC3967529

[12] Jegatheeswaran S, Siriwardena AK (2011) Experimental and clinical evidence for modification of hepatic ischaemia-reperfusion injury by N-acetylcysteine during major liver surgery. HPB (Oxford) 13: 71–78. 21241423 10.1111/j.1477-2574.2010.00263.x PMC3044340

[13] Newsholme P, Gaudel C, Krause M (2012) Mitochondria and diabetes. An intriguing pathogenetic role. Adv Exp Med Biol 942: 235–247. 10.1007/978-94-007-2869-1_10 22399425

[14] Coyle LC, Rodriguez A, Jeschke RE, Simon-Lee A, Abbott KC, Taylor AJ (2006) Acetylcysteine In Diabetes (AID): a randomized study of acetylcysteine for the prevention of contrast nephropathy in diabetics. Am Heart J 151: 1032 e1039–1012. 10.1016/j.ahj.2006.02.002 16644332

[15] Jaffery Z, Verma A, White CJ, Grant AG, Collins TJ, Grise MA, et al (2012) A randomized trial of intravenous n-acetylcysteine to prevent contrast induced nephropathy in acute coronary syndromes. Catheter Cardiovasc Interv 79: 921–926. 10.1002/ccd.23157 21542122

[16] Drummond GR, Selemidis S, Griendling KK, Sobey CG (2011) Combating oxidative stress in vascular disease: NADPH oxidases as therapeutic targets. Nat Rev Drug Discov 10: 453–471. 10.1038/nrd3403 21629295 PMC3361719

[17] Gao YJ, Ji RR (2010) Chemokines, neuronal-glial interactions, and central processing of neuropathic pain. Pharmacol Ther 126: 56–68. 10.1016/j.pharmthera.2010.01.002 20117131 PMC2839017

[18] Yuzefovych L, Wilson G, Rachek L (2010) Different effects of oleate vs. palmitate on mitochondrial function, apoptosis, and insulin signaling in L6 skeletal muscle cells: role of oxidative stress. Am J Physiol Endocrinol Metab 299: E1096–1105. 10.1152/ajpendo.00238.2010 20876761 PMC3006254

[19] Diaz-Vivancos P, de Simone A, Kiddle G, Foyer CH (2015) Glutathione—linking cell proliferation to oxidative stress. Free Radic Biol Med 89: 1154–1164. 10.1016/j.freeradbiomed.2015.09.023 26546102

[20] Marionneau C, Abriel H (2015) Regulation of the cardiac Na+ channel NaV1.5 by post-translational modifications. J Mol Cell Cardiol 82: 36–47. 10.1016/j.yjmcc.2015.02.013 25748040

[21] Zsurka G, Kunz WS (2015) Mitochondrial dysfunction and seizures: the neuronal energy crisis. Lancet Neurol 14: 956–966. 10.1016/S1474-4422(15)00148-9 26293567

[22] Gehrig SM, van der Poel C, Sayer TA, Schertzer JD, Henstridge DC, Church JE, et al (2012) Hsp72 preserves muscle function and slows progression of severe muscular dystrophy. Nature 484: 394–398. 10.1038/nature10980 22495301

[23] Sarszegi Z, Bognar E, Gaszner B, Konyi A, Gallyas F Jr., Sumegi B, et al (2012) BGP-15, a PARP-inhibitor, prevents imatinib-induced cardiotoxicity by activating Akt and suppressing JNK and p38 MAP kinases. Mol Cell Biochem 365: 129–137. 10.1007/s11010-012-1252-8 22350755

[24] Nagy G, Szarka A, Lotz G, Doczi J, Wunderlich L, Kiss A, et al (2010) BGP-15 inhibits caspase-independent programmed cell death in acetaminophen-induced liver injury. Toxicol Appl Pharmacol 243: 96–103. 10.1016/j.taap.2009.11.017 19931551

[25] Bardos G, Moricz K, Jaszlits L, Rabloczky G, Tory K, Racz I, et al (2003) BGP-15, a hydroximic acid derivative, protects against cisplatin- or taxol-induced peripheral neuropathy in rats. Toxicol Appl Pharmacol 190: 9–16. 12831778 10.1016/s0041-008x(03)00155-8

[26] Racz I, Tory K, Gallyas F Jr., Berente Z, Osz E, Jaszlits L, et al (2002) BGP-15—a novel poly(ADP-ribose) polymerase inhibitor—protects against nephrotoxicity of cisplatin without compromising its antitumor activity. Biochem Pharmacol 63: 1099–1111. 11931842 10.1016/s0006-2952(01)00935-2

[27] Literati-Nagy Z, Tory K, Literati-Nagy B, Kolonics A, Torok Z, Gombos I, et al (2012) The HSP co-inducer BGP-15 can prevent the metabolic side effects of the atypical antipsychotics. Cell Stress Chaperones 17: 517–521. 10.1007/s12192-012-0327-5 22322357 PMC3368033

[28] Gombos I, Crul T, Piotto S, Gungor B, Torok Z, Balogh G, et al (2011) Membrane-lipid therapy in operation: the HSP co-inducer BGP-15 activates stress signal transduction pathways by remodeling plasma membrane rafts. PLoS One 6: e28818 10.1371/journal.pone.0028818 22174906 PMC3236211

[29] Chung J, Nguyen AK, Henstridge DC, Holmes AG, Chan MH, Mesa JL, et al (2008) HSP72 protects against obesity-induced insulin resistance. Proc Natl Acad Sci U S A 105: 1739–1744. 10.1073/pnas.0705799105 18223156 PMC2234214

[30] Sapra G, Tham YK, Cemerlang N, Matsumoto A, Kiriazis H, Bernardo BC, et al (2014) The small-molecule BGP-15 protects against heart failure and atrial fibrillation in mice. Nat Commun 5: 5705 10.1038/ncomms6705 25489988

[31] Literati-Nagy B, Peterfai E, Kulcsar E, Literati-Nagy Z, Buday B, Tory K, et al (2010) Beneficial effect of the insulin sensitizer (HSP inducer) BGP-15 on olanzapine-induced metabolic disorders. Brain Res Bull 83: 340–344. 10.1016/j.brainresbull.2010.09.005 20849938

[32] Literati-Nagy Z, Tory K, Literati-Nagy B, Kolonics A, Vigh L Jr., Vigh L, et al (2012) A novel insulin sensitizer drug candidate-BGP-15-can prevent metabolic side effects of atypical antipsychotics. Pathol Oncol Res 18: 1071–1076. 10.1007/s12253-012-9546-4 22743983

[33] Galloway CA, Yoon Y (2013) Mitochondrial morphology in metabolic diseases. Antioxid Redox Signal 19: 415–430. 10.1089/ars.2012.4779 22793999 PMC3700066

[34] Hogeboom GH, Schneider WC (1950) Cytochemical studies of mammalian tissues. III. Isocitric dehydrogenase and triphosphopyridine nucleotide-cytochrome c reductase of mouse liver. J Biol Chem 186: 417–427. 14794638

[35] Sims NR (1990) Rapid isolation of metabolically active mitochondria from rat brain and subregions using Percoll density gradient centrifugation. J Neurochem 55: 698–707. 2164576 10.1111/j.1471-4159.1990.tb04189.x

[36] Papazisis KT, Geromichalos GD, Dimitriadis KA, Kortsaris AH (1997) Optimization of the sulforhodamine B colorimetric assay. J Immunol Methods 208: 151–158. 9433470 10.1016/s0022-1759(97)00137-3

[37] Tao H, Zhang Y, Zeng X, Shulman GI, Jin S (2014) Niclosamide ethanolamine-induced mild mitochondrial uncoupling improves diabetic symptoms in mice. Nat Med 20: 1263–1269. 10.1038/nm.3699 25282357 PMC4299950

[38] Teshima Y, Takahashi N, Nishio S, Saito S, Kondo H, Fukui A, et al (2014) Production of reactive oxygen species in the diabetic heart. Roles of mitochondria and NADPH oxidase. Circ J 78: 300–306. 24334638 10.1253/circj.cj-13-1187

[39] Robinson KM, Janes MS, Beckman JS (2008) The selective detection of mitochondrial superoxide by live cell imaging. Nat Protoc 3: 941–947. 10.1038/nprot.2008.56 18536642

[40] Ni R, Cao T, Xiong S, Ma J, Fan GC, Lacefield JC, et al (2016) Therapeutic inhibition of mitochondrial reactive oxygen species with mito-TEMPO reduces diabetic cardiomyopathy. Free Radic Biol Med 90: 12–23. 10.1016/j.freeradbiomed.2015.11.013 26577173 PMC5066872

[41] Huang LS, Cobessi D, Tung EY, Berry EA (2005) Binding of the respiratory chain inhibitor antimycin to the mitochondrial bc1 complex: a new crystal structure reveals an altered intramolecular hydrogen-bonding pattern. J Mol Biol 351: 573–597. 10.1016/j.jmb.2005.05.053 16024040 PMC1482829

[42] Klegeris A, McGeer PL (2001) Inflammatory cytokine levels are influenced by interactions between THP-1 monocytic, U-373 MG astrocytic, and SH-SY5Y neuronal cell lines of human origin. Neurosci Lett 313: 41–44. 11684335 10.1016/s0304-3940(01)02251-0

[43] von Bernhardi R, Eugenin-von Bernhardi L, Eugenin J (2015) Microglial cell dysregulation in brain aging and neurodegeneration. Front Aging Neurosci 7: 124 10.3389/fnagi.2015.00124 26257642 PMC4507468

[44] Brenner C, Galluzzi L, Kepp O, Kroemer G (2013) Decoding cell death signals in liver inflammation. J Hepatol 59: 583–594. 10.1016/j.jhep.2013.03.033 23567086

[45] Fonai F, Priber JK, Jakus PB, Kalman N, Antus C, Pollak E, et al (2015) Lack of cyclophilin D protects against the development of acute lung injury in endotoxemia. Biochim Biophys Acta 1852: 2563–2573. 10.1016/j.bbadis.2015.09.004 26385159

[46] Priber J, Fonai F, Jakus PB, Racz B, Chinopoulos C, Tretter L, et al (2015) Cyclophilin D disruption attenuates lipopolysaccharide-induced inflammatory response in primary mouse macrophages. Biochem Cell Biol 93: 241–250. 10.1139/bcb-2014-0120 25728038

[47] Tucsek Z, Radnai B, Racz B, Debreceni B, Priber JK, Dolowschiak T, et al (2011) Suppressing LPS-induced early signal transduction in macrophages by a polyphenol degradation product: a critical role of MKP-1. J Leukoc Biol 89: 105–111. 10.1189/jlb.0610355 20884647

[48] Seeley EJ, Rosenberg P, Matthay MA (2013) Calcium flux and endothelial dysfunction during acute lung injury: a STIMulating target for therapy. J Clin Invest 123: 1015–1018. 10.1172/JCI68093 23434597 PMC3582152

[49] Idelman G, Smith DL, Zucker SD (2015) Bilirubin inhibits the up-regulation of inducible nitric oxide synthase by scavenging reactive oxygen species generated by the toll-like receptor 4-dependent activation of NADPH oxidase. Redox Biol 5: 398–408. 10.1016/j.redox.2015.06.008 26163808 PMC4506991

[50] Gao L, Laude K, Cai H (2008) Mitochondrial pathophysiology, reactive oxygen species, and cardiovascular diseases. Vet Clin North Am Small Anim Pract 38: 137–155, vi. 10.1016/j.cvsm.2007.10.004 18249246 PMC2700041

[51] Jheng HF, Tsai PJ, Guo SM, Kuo LH, Chang CS, Su IJ, et al (2012) Mitochondrial fission contributes to mitochondrial dysfunction and insulin resistance in skeletal muscle. Mol Cell Biol 32: 309–319. 10.1128/MCB.05603-11 22083962 PMC3255771

[52] Circu ML, Aw TY (2010) Reactive oxygen species, cellular redox systems, and apoptosis. Free Radic Biol Med 48: 749–762. 10.1016/j.freeradbiomed.2009.12.022 20045723 PMC2823977

[53] Babot M, Birch A, Labarbuta P, Galkin A (2014) Characterisation of the active/de-active transition of mitochondrial complex I. Biochim Biophys Acta 1837: 1083–1092. 10.1016/j.bbabio.2014.02.018 24569053 PMC4331042

[54] Ray PD, Huang BW, Tsuji Y (2012) Reactive oxygen species (ROS) homeostasis and redox regulation in cellular signaling. Cell Signal 24: 981–990. 10.1016/j.cellsig.2012.01.008 22286106 PMC3454471

[55] Matsuzawa A, Ichijo H (2008) Redox control of cell fate by MAP kinase: physiological roles of ASK1-MAP kinase pathway in stress signaling. Biochim Biophys Acta 1780: 1325–1336. 10.1016/j.bbagen.2007.12.011 18206122

[56] Matsuno K, Iwata K, Matsumoto M, Katsuyama M, Cui W, Murata A, et al (2012) NOX1/NADPH oxidase is involved in endotoxin-induced cardiomyocyte apoptosis. Free Radic Biol Med 53: 1718–1728. 10.1016/j.freeradbiomed.2012.08.590 22982050

[57] Scott AJ, O'Dea KP, O'Callaghan D, Williams L, Dokpesi JO, Tatton L, et al (2011) Reactive oxygen species and p38 mitogen-activated protein kinase mediate tumor necrosis factor alpha-converting enzyme (TACE/ADAM-17) activation in primary human monocytes. J Biol Chem 286: 35466–35476. 10.1074/jbc.M111.277434 21865167 PMC3195581

[58] Korbecki J, Baranowska-Bosiacka I, Gutowska I, Chlubek D (2013) The effect of reactive oxygen species on the synthesis of prostanoids from arachidonic acid. J Physiol Pharmacol 64: 409–421. 24101387

